# Chronic Kidney Disease: Highlights for the General Pediatrician

**DOI:** 10.1155/2012/943904

**Published:** 2012-07-05

**Authors:** Raymond Quigley

**Affiliations:** Division of Pediatric Nephrology, UT Southwestern Medical Center, 5323 Harry Hines Boulevard, Dallas, TX 75390, USA

## Abstract

Chronic kidney disease in the pediatric population has been increasing. Early detection and treatment can slow down the progression of kidney disease and help prevent the development of end stage renal disease. In addition, as the kidney function declines, there are many pathophysiologic interactions with other organ systems that need to be monitored and treated. In particular, because of impaired vitamin D metabolism, calcium and phosphorus homeostasis is dysregulated and results in secondary bone disease. Anemia is common due to a number of factors including impaired erythropoietin production. Growth is often impacted by chronic kidney disease but can be improved by proper treatment. Complications of chronic kidney disease can be minimized by proper monitoring and treatment of these parameters. The general pediatrician plays a critical role in this process.

## 1. Introduction

Chronic kidney disease (CKD) had originally been defined as a glomerular filtration rate less than 60 mL/minute/ 1.73 m^2^ for a duration of 3 months or longer. This distinguished chronic kidney disease from episodes of acute kidney injury. For purposes of classification and treatment, the National Kidney Foundation developed a staging system for CKD based upon the patient's glomerular filtration rate (Table 1) [1]. Most of the data regarding the epidemiology and etiology of chronic kidney disease is based upon the adult population. However, there have been some studies recently that have begun to examine the epidemiology and etiology of chronic kidney disease in the pediatric population [2, 3]. This paper will examine the pathophysiology and epidemiology of chronic kidney disease in pediatrics. We will discuss the workup and management of these children from the perspective of a general pediatrician.

## 2. Pathophysiology

As the kidney function in the patient deteriorates, there are a number of pathophysiologic problems that develop in the patient. These will be reviewed according to the various organ systems that are affected. It will be important to consider the stage of chronic kidney disease that patient is in when thinking about these disorders.

One of the first problems that develop is related to bone disease [4]. The kidney plays a crucial role in activating vitamin D. The liver performs the 25-hydroxylation function, and the kidney performs the 1-alpha hydroxylation step. The 1,25-dihydroxy vitamin D that is formed is the most active form of vitamin D and will maintain healthy bones and prevent rickets in the growing child. Depending on the form of kidney disease the 1-alpha-hydroxylase function can begin to deteriorate at stage II or stage III chronic kidney disease. The patient can then develop hypocalcemia because of the decreased absorption of calcium in the gut. This will then lead to secondary hyperparathyroidism which will cause calcium to be mobilized from the bone. Some patients will actually present with pathologic fractures or other forms of bone disease as the presenting feature of chronic kidney disease.

In addition to the problems with calcium metabolism, as the glomerular filtration rate declines, the patients will also retain phosphorus and become hyperphosphatemic. This has been shown to stimulate fibroblast growth factor 23 which can lead to additional problems. A number of studies have demonstrated that early control of the patient's phosphate can alleviate many of the problems seen with chronic kidney disease. However, this can be very difficult to accomplish because the patient's dietary habits are beyond our control.

The interdependence of vitamin D, calcium, phosphorus and PTH is very complex [4]. The primary stimuli for PTH secretion are low ionized calcium and high serum phosphorus concentration. One of the actions of PTH is to stimulate the 1-alpha-hydroxylase enzyme in the renal cortex to activate more vitamin D. Vitamin D will then feed back to the parathyroid gland to decrease secretion of PTH. Vitamin D will also promote absorption of calcium and phosphorus from the intestines to help with mineralization of new bone. If the parathyroid gland is stimulated for a prolonged period of time by low calcium and high phosphorus, it will become autonomous and no longer be controlled by vitamin D. This is known as tertiary hyperparathyroidism.

Another area that is affected by chronic kidney disease is the patient's hemoglobin concentration [5]. As the patient's kidney function deteriorates, its ability to produce and secrete erythropoietin becomes impaired. In addition, as the patient becomes more uremic, the red cell half-life will decrease and so that turnover of the red cells will become increased. This can be corrected by treating the patients with exogenous erythropoietin. It is also crucial to make sure that patients do not become iron deficient. Prior to the availability of the erythropoietin, many of the chronic kidney disease patients would become iron overloaded because of the need for chronic transfusions. Now that these patients are treated with erythropoietin, many will become iron deficient.

The development of anemia is also linked with the problem of bone disease. If the patient's bone disease becomes advanced, they can develop a condition known as osteitis fibrosis cystica. In this condition the bone marrow becomes replaced with fibrous tissue and thus will not be able to respond to erythropoietin and cannot increase red cell production. Thus it becomes imperative to view the patient as a whole and treat both bone disease and anemia concomitantly.

A number of other electrolyte abnormalities come into play as the patient develops worsening chronic kidney disease. Oftentimes the patient will retain salt and develop hypertension. Potassium is normally secreted by the kidney but will oftentimes become a problem as the patient develops worsening renal function. The kidney is also responsible for maintaining the patient's acid base status by secreting protons. As the kidney function worsens, the patients often become more and more acidotic. This can lead to a number of problems such as worsening of the bone disease because the acidosis will enhance calcium mobilization from the bones and will worsen the bone disease.

As mentioned above, the retention of salt will cause trouble with hypertension. Also in many disease states, the kidney will be secreting renin that will also exacerbate blood pressure problems. More recently there is evidence that renal nerves play a role in the increased blood pressure in patients with chronic kidney disease. The elevated blood pressure will also cause damage to the kidneys and will accelerate the decline in renal function [6, 7]. In addition, the fluid retention could result in edema formation, both peripheral as well as pulmonary. As with adults with chronic kidney disease, hypertension causes significant cardiac problems in the pediatric population. This has become quite a focus in the treatment of these patients [6, 7]. It is imperative to maintain them at a normal blood pressure and prevent the cardiac problems that they could develop. There is also some evidence that parathyroid hormone and FGF 23 can cause some cardiac problems [4]. So as we discussed above with the bone disease and anemia, it appears that there is an interrelationship between the calcium and phosphorus metabolism and cardiac disease. 

Also more recently, a problem has been described with having elevated calcium and phosphorus simultaneously. This leads to an elevation in the calcium-phosphorus cross product and causes precipitation of calcium phosphate in the soft tissues. This has been shown to cause narrowing of the coronary arteries in adults and is becoming evident that this is a problem in pediatrics.

Patients with chronic kidney disease in general do not grow well [8]. Growth in these patients is a very complex problem involving many aspects of chronic kidney disease. As can be seen above, these patients with CKD have bone disease that will limit their growth potential. The fact that they are at risk for the development of cardiac disease probably also contributes to their growth problems. More importantly, as patients have declining renal function their appetite is suppressed. So many patients do not grow well because of poor nutrition. Other contributing factors include acidosis which impacts bone growth and chronic anemia, which can impact many factors including cardiac function and appetite.

Even when nutrition and bone disease are adequately addressed, patients with renal disease still may not grow well. It has been shown that the growth hormone-IGF 1 axis is abnormal in these patients. This is probably related to the interaction of IGF 1 and its binding protein. So after these patients have been treated with adequate nutrition and their bone disease is under control, they can be treated with growth hormone to stimulate growth [8].

## 3. Epidemiology

The incidence of chronic kidney disease (CKD) continues to increase in the United States. Unfortunately we do not have much data on the incidence or prevalence of chronic kidney disease and pediatric patients. Recently a few studies have initiated this, but it will take some time to get more data on this. A study that was published in 2003 reported on 4,666 pediatric patients with chronic kidney disease [3]. These patients were from North America, and the data was collected from centers specialized in pediatric nephrology. Most likely this number underrepresents the total number of chronic kidney disease patients in North America. So it remains difficult to know what the prevalence and incidence of chronic kidney disease are in the pediatric population.

## 4. Clinical Presentation 

In pediatric patients there are a number of causes of chronic kidney disease [3]. As opposed to adults, many pediatric patients develop chronic kidney disease secondary to congenital abnormalities in the urinary system. These patients are much more likely to have been followed more carefully after birth. In addition, there is more evidence that acute kidney injury can lead to chronic kidney disease [9–11]. Thus the clinical presentation will vary greatly depending on the cause of the chronic kidney disease.

There was some concern that urinary tract infections could eventually lead to chronic kidney disease. A recent study examined this question and showed that the infections themselves probably do not lead to chronic kidney disease [12]. The patients who develop chronic kidney disease had an abnormality demonstrated on a renal ultrasound or voiding cystourethrogram that predisposed the patient to urinary tract infections. The patients who had no defect did not progress to chronic kidney disease.

As discussed above oftentimes the patients will present with signs or symptoms secondary to anemia or bone disease. Thus when these patients are diagnosed, they already have significant chronic kidney disease causing problems.

In many patients the presentation of CKD can be more subtle. Many patients do not have an easily identifiable congenital problem. They may present with signs or symptoms that are not readily seen as referring to the renal system. These patients often have poor growth or chronic hypertension. In addition, if a patient has persistent proteinuria and hematuria, they should be evaluated for CKD.

## 5. Management

In terms of the workup for patients with chronic kidney disease, they need a very thorough assessment of their metabolic status. One of the primary laboratory tests to do is the serum creatinine. In a steady state this is one of the more common ways of estimating the renal function. While there are some problems with using the serum creatinine to estimate the renal function, it remains the main stay of clinical medicine [13, 14].

Other laboratory tests that need to be done include serum electrolytes to assess for hyperkalemia as well as the bicarbonate to determine if the patient is acidotic. The calcium and phosphorus also need to be monitored as well as parathyroid hormone and vitamin D level. Assessment of albumin will help monitor their nutritional status.

The need for imaging will depend upon the cause of the renal disease. Since many of the patients have obstruction as a cause of their chronic kidney disease, they may require periodic assessment with ultrasounds or possibly VCUGs. On initial evaluation, it is also important to examine a chest X-ray to evaluate the heart size. The patients might have significant left ventricular hypertrophy or they may have developed uremic pericarditis with a large pericardial effusion. If the patients complain about bone disease, they may need X-rays of their long bones and possibly bone densitometry performed.

Other testing that might need to be done includes a more accurate measure of the glomerular filtration rate. As discussed, above the serum creatinine may or may not give an accurate estimate of the patient's GFR. If a more accurate measure as needed, the patients can have their GFR measured using iothalamate or iohexol disappearance [15]. This test is performed by administering radioactive iothalamate to the patient, then measuring its disappearance from the blood stream and its appearance in the urine. This is a very accurate measure of the patient's glomerular filtration rate and is not dependent on the patient's muscle mass.

A number of chronic kidney diseases also involve the eyes and ears. So the patient might need a careful eye exam. For example patients with cystinosis will develop cystine crystals in the cornea. Many of the rheumatological diseases that cause chronic glomerulonephritis can also present with uveitis. Some other forms of nephronophthisis are associated with retinitis pigmentosa. Alport's syndrome which causes chronic kidney disease will cause lenticonus. Thus it is very important get a careful eye exam performed in patients that have chronic kidney disease. In addition Alport's will lead to high-frequency hearing loss.

## 6. Treatment

The treatment of patients with chronic kidney disease is focused on a number of areas [16]. Depending on the cause of the chronic kidney disease, the underlying disease might require specific treatment. For example, patients with cystinosis need to be on cysteamine to help prevent accumulation of cystine which will exacerbate the renal disease as well as cause other problems with the patient. Because many patients develop chronic kidney disease from obstructive uropathy, it will be important to make sure that their obstruction is relieved and they do not have ongoing problems with urinary tract infections.

Otherwise the treatment of these patients with chronic kidney disease will be aimed at controlling the blood pressure, the bone disease and their anemia. There are ample data now examining the effects of blood pressure and the development of cardiac disease in pediatrics [7, 17, 18]. Because many times patients have renin-mediated hypertension, it is best to control the blood pressure with an ACE inhibitor or an angiotensin receptor blocker. There is also more evidence that aldosterone may play a role in the cardiac disease in these patients. It may be beneficial to treat them with an aldosterone receptor blocker as well.

As discussed above, the metabolic bone disease, in these patients can be very significant. These patients develop hypovitaminosis D at an early stage of chronic kidney disease. Thus one of the early treatments will be supplementing their vitamin D. In the past this was done with activated 1,25-dihydroxy vitamin D or calcitriol. However more recently it has become evident that supplementing with 25-hydroxyvitamin D may also prove beneficial.

If the patients develop hyperkalemia, they may need to be treated with Kayexalate to help remove potassium from them. In infants who are dependent on being formula fed, the Kayexalate can be added to the formula. After the kayexalate has been thoroughly mixed with the formula, it will settle out and the formula can then be decanted. This way, the patient does not actually take the Kayexalate but receives the benefit of its use. Another way to help prevent hyperkalemia is to treat the patients with Lasix. This will of course depend on how much renal function the patient has and whether or not they will respond to a diuretic.

The long-term followup of these patients will of course depend on the stage of chronic kidney disease they have and how quickly they develop end-stage renal disease. If the patient has been optimally treated for the bone disease and has not responded well to nutritional support and growth hormone, they may need to have dialysis initiated to improve their growth and development. Ultimately these patients will do best with a kidney transplant. It is interesting to note that transplantation does not alleviate all of the problems related to chronic kidney disease. For example, it is known that they can continue to have significant bone disease after transplantation.

## 7. The Disease from a GP's Perspective

As with many subspecialty problems and pediatrics, there needs to be good communication between the general practitioner and the pediatric nephrologist. One of the issues with this has to do with the fact that most pediatric nephrologists are in large tertiary care centers. Thus many patients may have to travel quite a distance to see the nephrologist. It will improve the patient's care if the pediatrician can provide some of the local support.

For example, monitoring the patient's blood pressure can be done in the pediatrician's office, and if changes need to be made, that could be discussed with the nephrologist. Also many times the blood chemistries can be measured at the pediatrician's office and the results can be discussed with the nephrologist. This way the patient does not have to travel extensively for minor adjustments in their care.

In terms of diagnosing patients with chronic kidney disease, there are a number of things that can be addressed. Screening urinalyses have become somewhat controversial. If the patient is found to have microscopic hematuria, this might lead to a workup that is not productive. However, the presence of proteinuria seems to carry more significance. Thus one of the early signs of chronic kidney disease is the development of proteinuria. The pediatrician must keep in mind, however, that other common causes of proteinuria include orthostatic proteinuria and transient proteinuria that can occur during many illnesses.

As with many illnesses, the family history can be extremely important. Many patients with Alport's syndrome can be suspected from their family history. Autosomal dominant polycystic kidney disease is another very common cause of chronic disease in the adult population. This is also seen in many pediatric centers.

In addition to the family history and the urinalysis, it is important to monitor the blood pressure of patients. Oftentimes one of the first signs of serious kidney disease is the development of hypertension. So it would be very helpful if general pediatricians can measure the blood pressure and follow the guidelines set up in published in the 4th report [19].

## 8. Conclusion

The role of the general pediatrician in patients with chronic kidney disease can be very important. The general pediatrician can help identify patients at risk for chronic kidney disease early in the course of their disease. They can also participate in their ongoing care by being a resource. It is also important to remember that many subspecialists may not be adequately prepared to care for the other significant problems the patient might develop. So the general pediatrician can serve as a great resource to coordinate all of the care for the patient.

## Figures and Tables

**Table 1:** Stages of CKD as related to the GFR of the patient.

Chronic kidney disease stage	GFR (mL/min/1.73 m^2^)	
I	>90	Mild
II	60–90	Moderate
III	30–60	Moderate
IV	15–30	Severe
V	<15	ESRD

## References

[1] (2002). Part 4. Definition and classification of stages of chronic kidney disease. *American Journal of Kidney Diseases*.

[2] Warady BA, Chadha V (2007). Chronic kidney disease in children: the global perspective. *Pediatric Nephrology*.

[3] Seikaly MG, Ho PL, Emmett L, Fine RN, Tejani A (2003). Chronic renal insufficiency in children: the 2001 Annual Report of the NAPRTCS. *Pediatric Nephrology*.

[4] Schmitt CP, Mehls O (2011). Mineral and bone disorders in children with chronic kidney disease. *Nature Reviews Nephrology*.

[5] Atkinson MA, Furth SL (2011). Anemia in children with chronic kidney disease. *Nature Reviews Nephrology*.

[6] Wuhl E, Schaefer F (2010). Can we slow the progression of chronic kidney disease?. *Current Opinion in Pediatrics*.

[7] Wühl E, Schaefer F (2011). Managing kidney disease with blood-pressure control. *Nature Reviews Nephrology*.

[8] Rees L, Mak RH (2011). Nutrition and growth in children with chronic kidney disease. *Nature Reviews Nephrology*.

[9] Goldstein SL, Devarajan P (2008). Progression from acute kidney injury to chronic kidney disease: a pediatric perspective. *Advances in Chronic Kidney Disease*.

[10] López-Novoa JM, Rodríguez-Peña AB, Ortiz A, Martínez-Salgado C, López Hernández FJ (2011). net Etiopathology of chronic tubular, glomerular and renovascular nephropathies: clinical implications. *Journal of Translational Medicine*.

[11] Murugan R, Kellum JA (2011). Acute kidney injury: what’s the prognosis?. *Nature Reviews Nephrology*.

[12] Salo J, Ikäheimo R, Tapiainen T, Uhari M (2011). Childhood urinary tract infections as a cause of chronic kidney disease. *Pediatrics*.

[13] Uhlig K, Balk EM, Lau J, Levey AS (2006). Clinical practice guidelines in nephrology—for worse or for better. *Nephrology Dialysis Transplantation*.

[14] Seikaly MG, Browne R, Bajaj G, Arant BS (1996). Limitations to body length/serum creatinine ratio as an estimate of glomerular filtration in children. *Pediatric Nephrology*.

[15] Bajaj G, Alexander SR, Browne R, Sakarcan A, Seikaly MG (1996). 125Iodine-iothalamate clearance in children. A simple method to measure glomerular filtration. *Pediatric Nephrology*.

[16] Hogg RJ, Furth S, Lemley KV (2003). National Kidney Foundation’s Kidney Disease Outcomes Quality Initiative clinical practice guidelines for chronic kidney disease in children and adolescents: evaluation, classification, and stratification. *Pediatrics*.

[17] Hilgers KF, Dötsch J, Rascher W, Mann JFE (2004). Treatment strategies in patients with chronic renal disease: ACE inhibitors, angiotensin receptor antagonists, or both?. *Pediatric Nephrology*.

[18] Shroff R, Weaver Jr. DJ, Mitsnefes MM (2011). Cardiovascular complications in children with chronic kidney disease. *Nature Reviews Nephrology*.

[19] Falkner B, Daniels SR, Flynn JT (2004). The fourth report on the diagnosis, evaluation, and treatment of high blood pressure in children and adolescents. *Pediatrics*.

